# Supplementary material for: Oxidized Phospholipids Regulate Tenocyte Function via Induction of Amphiregulin in Dendritic Cells
Source: Int J Mol Sci. 2024 Jul 11;25(14):7600. doi: 10.3390/ijms25147600 (PMC11277520; doi:10.3390/ijms25147600)
Supplement: Supplementary file 1 [file ijms-25-07600-s001.zip › ijms-3081006-supplementary.pdf]

Supplementary Figure S1

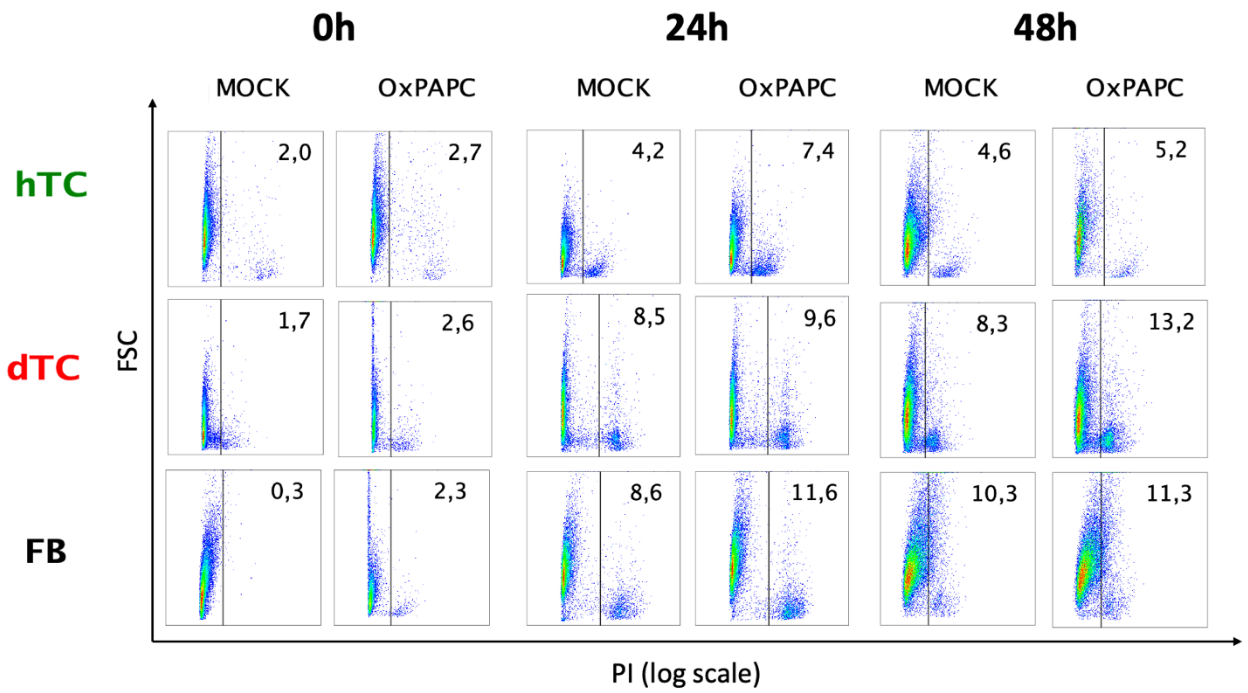

Treatment with OxPAPC is not cytotoxic for TC or FB. Viability of the cells was investigated by propidium iodide (PI) staining and analyzed via flow cytometry. Analyses of viable and dead (PI positive) cells upon treatment with or without (mock) OxPAPC (30 µg/ml) was performed at 0, 24 and 48h. The marker (black line) separates live from the dead cells and the percentage of dead cells is shown in the upper right corner of the dot-blots.

Supplementary Figure S2

A

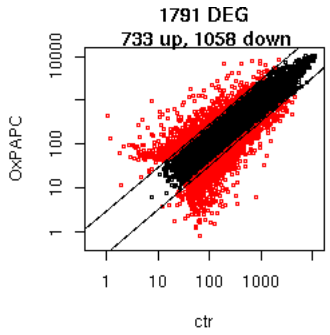

B

Symbol	*Name/Function*	*Fold Induction*
F2RL2	coagulation factor II (thrombin) receptor-like 2	430
SLC16A10	solute carrier family 16 (monocarboxylic acid transporters), member 10	130
AKR1C3	aldo-keto reductase family 1, member C3 (3-alpha hydroxysteroid dehydrogenase, type II)	114
THBS1	thrombospondin 1	76
KITLG	KIT ligand	68
AREG	amphiregulin (schwannoma-derived growth factor)	51
PPBP	pro-platelet basic protein (chemokine (C-X-C motif) ligand 7)	43
CXCL2	chemokine (C-X-C motif) ligand 2	42
CXCL3	chemokine (C-X-C motif) ligand 3	40
HSPA4L	heat shock 70kDa protein 4-like	38

Idendification of OxPAPC-induced genes in DCs. (A) RNA from unstimulated and OxPAPC-DC of 6 different donors was pooled and analyzed with the Affymetrix HG_U133Plus_2.0 GeneChip. The diagonal „line of identity“ indicates equal expression in control DC and OxPAPC- treated DC. Points outside the indicated region were statistically increased or downregulated. (B) In OxPAPC-treated DCs 1791 genes were differentially expressed relative to unstimulated cells (of which 733 genes were upregulated and 1058 genes were down-regulated. Amphiregulin (AREG) (51-fold induction) was amongst the 10 strongest upregulated genes.
